# Associations between mental health challenges, sexual activity, alcohol consumption, use of other psychoactive substances and use of COVID-19 preventive measures during the first wave of the COVID-19 pandemic by adults in Nigeria

**DOI:** 10.1186/s12889-023-16440-x

**Published:** 2023-08-09

**Authors:** Morenike Oluwatoyin Folayan, Olanrewaju Ibigbami, Maha El Tantawi, Nourhan M. Aly, Roberto Ariel Abeldaño Zuñiga, Giuliana Florencia Abeldaño, Eshrat Ara, Passent Ellakany, Balgis Gaffar, Nuraldeen Maher Al-Khanati, Ifeoma Idigbe, Anthonia Omotola Ishabiyi, Abeedha Tu-Allah Khan, Zumama Khalid, Folake Barakat Lawal, Joanne Lusher, Ntombifuthi P. Nzimande, Bamidele Olubukola Popoola, Mir Faeq Ali Quadri, Mark Roque, Joseph Chukwudi Okeibunor, Brandon Brown, Annie Lu Nguyen

**Affiliations:** 1 Mental Health and Wellness Study Group, Ile-Ife, Nigeria; 2 Faculty of Dentistry, Obafemi Awolowo University, Ile-Ife, Nigeria; 3 Department of Mental Health, Obafemi Awolowo University, Ile-Ife, Nigeria; 4 Department of Pediatric Dentistry and Dental Public Health, Faculty of Dentistry, Alexandria University, Alexandria, 21527 Egypt; 5 Postgraduate Department, University of Sierra Sur, Oaxaca, Mexico; 6 School of Medicine, University of Sierra Sur, Oaxaca, Mexico; 7 Department of Psychology, Government College for Women, Moulana Azad Road Srinagar Kashmir (Jammu and Kashmir), Srinagar, 190001 India; 8 College of Dentistry, Substitutive Dental Sciences, Imam Abdulrahman Bin Faisal University, Dammam, Saudi Arabia; 9 Department of Preventive Dental Sciences, College of Dentistry, Imam Abdulrahman Bin Faisal University, Dammam, Saudi Arabia; 10 Department of Oral and Maxillofacial Surgery, Faculty of Dentistry, Syrian Private University, Damascus, Syria; 11 Clinical Sciences Department, Nigerian Institute of Medical Research, Lagos, Nigeria; 12 Department of Sociology, University of Cincinnati, Cincinnati, OH USA; 13 School of Biological Sciences, University of the Punjab, Quaid-I-Azam Campus, Lahore, 54590 Pakistan; 14 Department of Periodontology and Community Dentistry, University of Ibadan and University College Hospital, Ibadan, Nigeria; 15 Provost’s Group, Regent’s University London, London, UK; 16 Department of Economic and Human Geography, Faculty of Geosciences, University of Szeged, 6722 Szeged, Hungary; 17 Department of Child Oral Health, University of Ibadan, Ibadan, Nigeria; 18 Division of Dental Public Health, Department of Preventive Dental Sciences, Jazan University, Jazan, Saudi Arabia; 19 Maternity and Childhood Department, College of Nursing, Taibah University, Madinah, Kingdom of Saudi Arabia; 20 WHO Regional Office for Africa, Brazzaville, BP 06 Congo; 21 Department of Social Medicine, Population and Public Health, Riverside School of Medicine, University of California, Riverside, CA USA; 22 Herbert Wertheim School of Public Health and Human Longevity Science, University of California, San Diego, CA USA

**Keywords:** Physical distancing, Sexual activity, Alcohol consumption, Anxiety, Depression, Post-traumatic stress disorder, SARS-CoV-2

## Abstract

**Background:**

The aims of this study were to assess: 1) the associations among sexual activity, alcohol consumption, use of other psychoactive substances and mental health during the COVID-19 pandemic; and 2) the associations between COVID-19 preventive measures, alcohol consumption and use of psychoactive substances.

**Methods:**

This was a secondary analysis of data collected from adults in Nigeria between July and December 2020. The variables extracted included change in sexual activity, alcohol consumption and use of other psychoactive substances, COVID-19 preventive behaviors (wearing face masks, washing hands, physical distancing), anxiety, depression, post-traumatic stress disorder (PTSD) and sociodemographic variables (age, sex, education, HIV status, employment status). Multivariable logistic regressions were conducted. A model was run to regress depression, anxiety, PTSD, increased alcohol consumption, and increased use of other psychoactive substances, on increased sexual activity. In separate models, anxiety, depression, and PTSD were regressed on increased alcohol consumption and on increased use of other psychoactive substances. Finally, three models were constructed to determine the associations between increased alcohol consumption and increased use of other psychoactive substances on three separate COVID-19 preventive behaviors. All models were adjusted for sociodemographic variables.

**Results:**

Increased alcohol consumption (AOR:2.19) and increased use of other psychoactive substances (AOR: 3.71) were significantly associated with higher odds of increased sexual activity. Depression was associated with significantly higher odds of increased alcohol consumption (AOR:1.71) and increased use of other psychoactive substances (AOR:3.21). Increased alcohol consumption was associated with significantly lower odds of physical distancing (AOR:0.59).

**Conclusion:**

There was a complex inter-relationship between mental health, sexual health, increased use of psychoactive substances. The consumption of alcohol also affected compliance with physical distancing. Further studies are needed to understand the observed relationships.

## Background

During the COVID-19 pandemic, lockdowns instituted in response to the public health emergency created economic losses and social disruptions that led to multiple direct and indirect health consequences [1]. One such consequence was the global increase in anxiety, depression, and post-traumatic stress disorder [2–4]. Concurrently, there were shifts in sexual behaviors [5–11]. Research has demonstrated greater sexual dysfunction to be associated with greater mental health challenges [12–17]. During the pandemic, frequency of sexual activity may be reduced due to restrictions in physical movement among couples who live in separate dwellings or geographical locations [13]. In the absence of sexual partners, individuals may engage in solitary sexual activities, which may cause emotional distress for people in certain cultures. In Nigeria, for example, solitary sexual activities are stigmatized by traditional religious and societal norms [18, 19]. Individuals with unmet needs for their sexual desires may also experience a perceived reduction in sexual and mental well-being [20–22].

Mental health challenges were also associated with increased use of psychoactive substances during the COVID-19 pandemic. In fact, increases in psychoactive substance use during the COVID-19 pandemic were reported in Australia [23], Brazil [24], Canada [25], Germany [26] and the United States [27]. Some individuals use substances, including alcohol, as a maladaptive coping strategy for reducing stress, maintaining a state of physical and mental relaxation, and improving social behavior [28–33]. However, high levels of psychoactive substance use can result in the inhibition of the central nervous system, reduce discernment, weaken attention and memory, resulting in increased risk for poor decision-making [34], anxiety and depression [35, 36]. Within the context of COVID-19, the confluence of altered decision-making capacity, anxiety and depression may serve to negatively impact the uptake and use of COVID-19 prevention measures [37].

There is limited empirical evidence on psychoactive substance use in sub-Saharan Africa during the pandemic, although general use of psychoactive substances by adolescents in sub-Saharan Africa is high. Up to 41.6% of adolescents use at least one psychoactive substance with 32.8% reporting consumption of alcohol and 3.9% reporting the use of cocaine [38]. In Nigeria, about 14.3 million people between ages 15 and 64 years used psychoactive substances for non-medical purposes in 2017 [39].

This study draws upon a conceptual framework that suggests mental health challenges (anxiety, depression, and post-traumatic stress disorder) during the COVID-19 pandemic may have an impact on sexual activities and the use of psychoactive substances in an African population, where cultural beliefs and social expectations may have otherwise resulted in restraints in sexual practices and psychoactive substance use. Using the Cognitive Escape Theory [40], we hypothesize that the cultural beliefs and social expectations that constrain sexual behaviors and use of psychoactive substances by individuals may become cognitively burdensome under the added mental stress (anxiety, depression, post-traumatic stress disorder) created by the COVID-19 pandemic, thereby motivating a behavioral “rebound” that allows individuals to escape from this constraint.

Cognitive restraint, when combined with expectations resulting from cultural beliefs and social expectations, can lead to strategic use of substances. Substances could be used for the purposes of lowering sexual inhibitions and/or enhancing sexual pleasure [41–43]. We therefore conceptualize an analytical framework that considers the relationship between sexual behaviors and its correlates (anxiety, depression, post-traumatic stress disorder, alcohol consumption and use of psychoactive substances) during the COVID-19 pandemic. Additionally, recognizing that COVID-related safety during the pandemic was paramount, we included COVID-19 behaviors as outcomes in the framework. Alcohol and psychoactive substances use increased in Nigeria because of the pandemic [44, 45] but the associations between the use of psychoactive substances and COVID-19 behaviors remains understudied in a population like Nigeria where risk-taking is high. The rationale for this study was to assess the complex relationships between these factors in an adult population in Nigeria during the COVID-19 pandemic, taking into consideration the cultural, social, sexual, psychological, and economic context of the country.

The first aim of this study was to examine the relationship among anxiety, depression, post-traumatic stress disorder (PTSD), consumption of alcohol and other psychoactive substances, and self-reported changes in sexual activity and during the COVID-19 pandemic. The second aim was to determine the associations between alcohol consumption and use of other psychoactive substances and COVID-19 preventive behaviors. We hypothesized that 1) increased anxiety, depression, PTSD, alcohol consumption, and use of psychoactive substances will be associated with self-reported changes in sexual activity; 2) greater anxiety, depression and PTSD will be associated with increased alcohol consumption and increased psychoactive substances use; and 3) increased alcohol consumption and increased use of psychoactive substances will be associated with non-adherence to COVID-19 preventive measures.

## Methods

The data used in this secondary analysis were obtained from a cross-sectional, multi-country study of 21,206 adults aged 18 years and older. Respondents resided across 152 countries and data were collected between July and December 2020 using an online survey platform (Survey Monkey ®). Details on the methodology of this study have been reported in prior publications [46–48]. Briefly, participants were recruited through non-probability, respondent-driven, sampling methods (exponential non-discriminatory snowballing and crowdsourcing) initiated by data collectors who shared the survey links on social media (Facebook, Twitter, Instagram, WhatsApp) and email lists. Data collectors also recruited members of their networks and respondents were encouraged to further share the link within their own networks. Participation was voluntary and consent was indicated by selecting a checkbox before proceeding to the questionnaire. The survey utilized a validated questionnaire to collect data describing COVID-19 on the mental health and wellness and wellbeing of respondents [49]. The overall Content Validity Index of the questionnaire was 0.83. The questionnaire took an average of 11 min to complete. Each participant could only complete a single questionnaire through IP address restrictions, though they could edit their answers freely until they chose to submit. No questions were made compulsory. Ethical approval was obtained from the Human Research Ethics Committee at the Institute of Public Health of the Obafemi Awolowo University Ile-Ife, Nigeria (HREC No: IPHOAU/12/1557).

Only the data from Nigerian respondents who had data for sexual activity, alcohol use and other psychoactive substance were obtained for the current study. Multiple best-practice procedures were performed to increase the data quality of the survey. Complete case analysis was employed [50]. Also, the data was checked to identify and remove any survey responses completed below seven minutes (N = 77)—the lower limit of the time range to answer the questionnaire during the pilot phase. The number of participants whose data were extracted for this study was considered statistically adequate for the research questions as there was a minimum of 10 participants with complete responses for each dependent variable available for this study. This enabled the performance of regression analyses with a minimum probability level of 0.05 [51, 52].

### Study variables

#### Sexual activity, alcohol consumption and use of other psychoactive substances

Participants were asked: Have you experienced a change in (a) sexual activity, (b) tobacco use, (c) alcohol use, (d) marijuana use, and (e) other substance use during the COVID-19 pandemic? Response options were: increase, decrease, no change or not applicable. [48]. Participants who indicated “not applicable” were considered not sexually active, not a consumer of alcohol or not a user of psychoactive substances and excluded from the dataset.

For the logistic regression analysis, sexual activity was dichotomized into increased versus no increase in sexual activity (combining no change and decreased for the latter category). Alcohol consumption was dichotomized into increased versus no increase in alcohol use (combining no change and decreased for the latter category). The use of other psychoactive substances was dichotomized into increased/no increase in use of other psychoactive substances (combining no change and decreased for the latter category).

#### COVID-19 prevention behaviors

Respondents were asked: Which of the following are you practicing during COVID-19? An endorsement indicated they had adopted that behavior during the pandemic. The options were: 1) wearing masks or face coverings, 2) washing my hands or sanitizing my hands more often, and 3) practicing physical distancing (i.e., reducing physical contact with other people in social, work, or school settings by avoiding large groups and staying 6 feet away from other people. A selection of the checkbox was treated as a “yes” response while a non-selection was treated as a “no” response. These questions were a component of the Pandemic Stress Index [53].

#### Post-traumatic stress disorder (PTSD)

The PTSD checklist for civilians was used to measure the level of PTSD in respondents. The checklist is a 17-item self-report questionnaire that measured symptoms of PTSD [54]. The questionnaire prompted respondents to measure the level of stress that they have pertaining to a problem or complaint in response to a stressful life experience (in this case the COVID-19 pandemic) over the past month. A 5-point Likert scale was used for respondents to rate their responses (1- not at all to 5- extremely). The possible scores ranged from 17–85. The recommended cut-off score of 44 for indicating potentially clinically relevant symptoms of PTSD in civilian primary care was used in this study [55]. The Cronbach alpha score for the tool for respondents from Nigeria was 0.949.

#### Anxiety and depression

The questions were part of the Pandemic Stress Index assessing the psychosocial impact of COVID-19 [55]. Respondents were asked: Which of the following are you experiencing during this COVID-19 period? Respondents were required to select all that applied on a list of 21 options. A selection on any item on the list was categorized as a “yes” response and absence of selection was categorized as a “no” response. For this study, the responses to anxiety and depression were extracted.

#### Sociodemographic profile

Data were collected on age, sex at birth, highest level of education attained (none, primary, secondary, college/university) and employment status (employed, unemployed, student, retired).

#### HIV status

A question was also asked about HIV status. Respondents were asked to identify their HIV status as positive, negative, unknown, or unwilling to declare. The respondents who identified as HIV positive were described as “living with HIV,” while the those who identified as HIV negative or unknown were described as “not living with HIV.” And those unwilling to declare were excluded from the study. HIV status was included as a confounding variable because of its associations with the dependent [56, 57] and independent variables [58–61] and because Nigeria is a country with high HIV burden, ranking third highest in the world [62].

#### Statistical analysis

Raw data were downloaded, cleaned, and imported to IBM SPSS® for Windows, Version 23.0 (Armonk, NY: IBM Corp) for analyses. Descriptive analysis of the study variables was conducted. Tests of associations (ANOVA and chi square test) were used to assess the relationship between the dependent variables (sexual activity, alcohol consumption and use of psychoactive substances) and the independent variables. Seven multivariable logistic regression models were built with different dependent variables (DV) and independent variables (IDV) as follows:

- Model 1: DV- increased sexual activity. IDV- mental health status** (**PTSD, anxiety, depression), increased alcohol consumption and increased use of other psychoactive substances.
- Model 2: DV- increased alcohol consumption during the pandemic. IDV -mental health status** (**PTSD, anxiety, depression**)**.
- Model 3: DV- increased use of other psychoactive substances during the pandemic. IDV- mental health status **(**PTSD, anxiety, depression**)**.
- Model 4: DV- use of face mask. IDV- increased alcohol consumption and increased use of other psychoactive substances.
- Model 5: DV- frequent hand washing. IDV- increased alcohol consumption and increased use of other psychoactive substances.
- Model 6: DV- practicing physical distancing. IDV- increased alcohol consumption and increased use of other psychoactive substances.

The models were adjusted for confounders (age, sex at birth, HIV status, highest level of education attained and employment status). Adjusted odds ratios (AORs) and 95% confidence intervals (CIs) were calculated. The Omnibus test of model coefficients was used to determine the significant difference between the Log-likelihoods (specifically the -2LLs) of the baseline models and the new models inclusive of the explanatory variable. Statistical significance was set at < 0.05.

## Results

Table 1 shows that the mean age of the respondents was 39.1 (SD = 10.8) years, 1,636 (51.8%) were males, 2,605 (82.4%) had college/ university education, and 2,373 (75.1%) were employed. Regarding mental health, 884 (28.0%) experienced PTSD, 603 (19.1%) experienced anxiety, and 310 (9.8%) experienced depression. Regarding the use of COVID-19 preventive measures, 2,662 (84.2%) reported wearing face masks, 2,592 (82%) reported frequent handwashing and 2,445 (77.4%) practiced physical distancing.

**Table 1:** Factors associated with changes in sexual activity, alcohol consumption and use of psychoactive substances during the COVID-19 pandemic by adults in Nigeria

Variables	Total n (%)	Sexual activity (*N* = 3160)			*p* value	Total n (%)	Alcohol consumption (*N* = 957)			*p* value	Total n (%)	Use of psychoactive substances (*N* = 260)			*p* value
												Decreased *N* = 54 n (%)	Increased *N* = 51 n (%)	No change *N* = 155 n (%)	
		Decreased *N* = 925 n (%)	Increased *N* = 596 n (%)	No change *N* = 1639 n (%)											
							Decreased *N* = 343 n (%)	Increased *N* = 182 n (%)	No change *N* = 432 n (%)						
Age in years Mean (SD)	39.1 (10.8)	39.5 (11.3)	37.2 (9.6)	39.6 (10.9)	< 0.001	37.9 (11.4)	37.1 (11.3)	36.4 (10.3)	39.3 (11.5)	0.002	33.8 (9.7)	34.6 (10.3)	33.9 (9.6)	33.3 (9.4)	0.654
Sex at birth															
Male	1636 (51.8)	496 (30.3)	321 (19.6)	819 (50.1)	*0.236	610 (63.7)	220 (36.1)	123 (20.2)	267 (43.8)	*0.177	152 (58.5)	36 (23.7)	31 (20.4)	85 (55.9)	*0.410
Female	1506 (47.7)	426 (28.3)	273 (18.1)	807 (53.6)		342 (35.7)	119 (34.8)	59 (17.3)	164 (48.0)		106 (40.8)	17 (16.0)	20 (18.9)	69 (65.1)	
Intersex	3 (0.1)	0 (0.0)	0 (0.0)	3 (100.0)		-	-	-	-		-	-	-	-	
Declined to answer	15 (0.5)	3 (20.0)	2 (13.3)	10 (66.7)		5 (0.5)	4 (80.0)	0 (0.0)	1 (20.0)		2 (0.8)	1 (5?0.0)	0 (0)	1 (50.0)	
Highest educational level															
No formal education	37 (1.2)	13 (35.1)	11 (29.7)	13 (35.1)	< 0.001	21 (2.2)	3 (14.3)	11 (52.4)	7 (33.3)	* < 0.001	4 (1.5)	0 (0.0)	2 (50.0)	2 (50.0)	*0.124
Primary	63 (2.0)	26 (41.3)	19 (30.2)	18 (28.6)		23 (2.4)	8 (34.8)	11 (47.8)	4 (17.4)		5 (1.9)	2 (40.0)	1 (20.0)	2 (40.0)	
Secondary	455 (14.4)	143 (31.4)	109 (24.0)	203 (44.6)		148 (15.5)	62 (41.9)	29 (19.6)	57 (38.5)		59 (22.7)	18 (30.5)	13 (22.0)	28 (47.5)	
College/university	2605 (82.4)	743 (28.5)	457 (17.5)	1405 (53.9)		765 (79.9)	270 (35.3)	131 (17.1)	364 (47.6)		192 (73.8)	34 (17.7)	35 (18.2)	123 (64.1)	
Employment Status															
Employed	2373 (75.1)	667 (28.1)	436 (18.4)	1270 (53.5)	0.001	139 (14.5)	62 (44.6)	24 (17.3)	53 (38.1)	0.246	48 (18.5)	11 (22.9)	12 (25.0)	25 (52.1)	0.018
Unemployed	459 (14.5)	156 (34.0)	90 (19.6)	213 (46.4)		710 (74.2)	237 (33.4)	139 (19.6)	334 (47.0)		172 (66.2)	29 (16.9)	34 (19.8)	109 (63.4)	
Student	260 (8.2)	74 (28.5)	65 (25.0)	121 (46.5)		83 (8.7)	33 (39.8)	15 (18.1)	35 (42.2)		35 (13.5)	10 (28.6)	5 (14.3)	20 (57.1)	
Retired	68 (2.2)	28 (41.2)	5 (7.4)	35 (51.5)		25 (2.6)	11 (44.0)	4 (16.0)	10 (40.0)		5 (1.9)	4 (80.0)	0 (0.0)	1 (20.0)	
*HIV status*															
Not living with HIV	2439 (77.2)	688 (28.2)	409 (16.8)	1342 (55.0)	< 0.001	730 (76.3)	248 (34.0)	131 (17.9)		0.005	201 (77.3)	38 (18.9)	35 (17.4)	128 (63.7)	0.047
Living with HIV	721 (22.8)	237 (32.9)	187 (25.9)	297 (41.2)		227 (23.7)	95 (41.9)	51 (22.5)			59 (22.7)	16 (27.1)	16 (27.1)	27 (45.8)	
Mental health status															
* PTSD*															
No	2276 (72.0)	591 (26.0)	393 (17.3)	1292 (56.8)	< 0.001	631 (65.9)	223 (35.3)	99 (15.7)	309 (49.0)	< 0.001	148 (56.9)	20 (13.5)	25 (16.9)	103 (69.6)	< 0.001
Yes	884 (28.0)	334 (37.8)	203 (23.0)	347 (39.3)		326 (34.1)	120 (36.8)	83 (25.5)	123 (37.7)		112 (43.1)	34 (30.4)	26 (23.2)	52 (46.4)	
* Anxiety*															
No	2557 (80.9)	673 (26.3)	471 (18.4)	1413 (55.3)	< 0.001	744 (77.7)	259 (34.8)	132 (17.7)	353 (47.4)	0.020	206 (79.2)	46 (22.3)	38 (18.4)	12 (59.2)	0.391
Yes	603 (19.1)	252 (41.8)	125 (20.7)	226 (37.5)		213 (22.3)	84 (39.4)	50 (23.5)	79 (37.1)		54 (20.8)	8 (14.8)	13 (24.1)	33 (61.1)	
* Depression*															
No	2850 (90.2)	795 (27.9)	514 (18.0)	1541 (54.1)	< 0.001	837 (87.5)	296 (35.4)	144 (17.2)	397 (47.4)	< 0.001	224 (86.2)	46 (20.5)	39 (17.4)	139 (62.1)	0.059
Yes	310 (9.8)	130 (41.9)	82 (26.5)	98 (31.6)		120 (12.5)	47 (39.2)	38 (31.7)	35 (29.2)		36 (13.8)	8 (22.2)	12 (33.3)	16 (44.4)	
Use of COVID-19 preventive measures															
* Face masks*															
No						197 (20.6)	74 (37.6)	40 (20.3)	83 (42.1)	0.632	89 (34.2)	26 (29.2)	16 (18.0)	47 (52.8)	0.053
Yes						760 (79.4)	269 (35.4)	142 (18.7)	349 (45.9)		171 (65.8)	28 (16.4)	35 (20.5)	108 (63.2)	
* Washing of hands/sanitizing*															
No						203 (21.2)	74 (36.5)	45 (22.2)	84 (41.4)	0.336	81 (31.2)	19 (23.5)	19 (23.5)	43 (53.1)	0.344
Yes						754 (78.8)	269 (35.7)	137 (18.2)	348 (46.2)		179 (68.8)	35 (19.6)	32 (17.9)	112 (62.6)	
* Physical distancing*															
No						249 (26.0)	86 (34.5)	69 (27.7)	94 (37.8)	< 0.001	78 (30.0)	20 (25.6)	19 (24.4)	39 (50.0)	0.118
Yes						708 (74.0)	257 (36.3)	113 (16.0)	338 (47.7)		182 (70.0)	34 (18.7)	32 (17.6)	116 (63.7)	

*Fishers exact test used

Of the 3,160 participants who reported been sexually active during the pandemic, 596 (18.9%) reported an increase in sexual activity and 925 (29.3%) reported a decrease during the COVID-19 pandemic. Respondents who reported an increase in sexual activity during the pandemic were significantly younger than those who reported a decrease or no change (*p* < 0.001). A higher percentage of participants with PTSD (*p* < 0.001), anxiety (*p* < 0.001) and depression (*p* < 0.001) reported changes in sexual activity compared to those who did not experience these conditions. There were no significant associations between sexual activity and the use of COVID-19 preventive measures.

Of the 957 participants who reported consuming alcohol, 343 (35.8%) decreased consumption and 182 (19.0%) increased consumption. About one-third (34.1%), experienced PTSD, 213 (22.3%) experienced anxiety and 120 (12.5%) experienced depression. Significantly more respondents with PTSD (*p* < 0.001), and those who experienced anxiety (*p* = 0.020) and depression (*p* < 0.001) increased alcohol consumption compared to those without those conditions. Also, a significantly greater number of respondents who did not practice physical distancing increased alcohol consumption (*p* < 0.001). Of the 260 participants who reported using other psychoactive substance, 54 (20.8%) decreased their use while 51 (19.6%) increased use. Also, 112 (43.1%) experienced PTSD, 54 (20.8%) experienced anxiety and 36 (13.8%) experienced depression. Significantly more respondents who had PTSD (*p* < 0.001) increased the use of other psychoactive substances. There was no significant association between use of other psychoactive substances and COVID-19 preventive measures.

Table 2 highlights the variables associated with changes in sexual activity, alcohol consumption and use of other psychoactive substances during the COVID-19 pandemic. The odds of increased sexual activity during the COVID-19 pandemic were significantly higher for those who increased alcohol consumption (AOR = 22.18; 95% CI: 1.54, 3.07; *p* < 0.001) and increased the use of other psychoactive substances (AOR = 3.67; 95% CI: 2.01, 6.73; *p* < 0.001). Furthermore, the odds of increased alcohol consumption during the COVID-19 pandemic were significantly higher among respondents who experienced PTSD (AOR = 1.50; 95% CI: 1.05, 2.82; *p* = 0.028), and depression (AOR = 1.72; 95% CI: 1.05, 2.82; *p* = 0.032).

**Table 2:** Multivariable logistic regression analyses on the associations between increased sexual activity, increased alcohol use, increased use of other psychoactive substances and mental health status during the COVID-19 pandemic by adults in Nigeria

Variables	Increased sexual activity (*N* = 3160)		Increased alcohol consumption (*N* = 957)		Increased use of other psychoactive substances (*N* = 247)	
	**AOR** **(95% CI)**	***p* value**	**AOR** **(95% CI)**	***p* value**	**AOR** **(95% CI)**	***p* value**
**Mental health status**						
* PTSD*						
Yes (ref: no PTSD)	1.15 (0.94–1.42)	0.180	1.50 (1.05–2.16)	0.028	0.72 (0.36–1.44)	0.356
* Anxiety*						
Yes (ref: No anxiety)	1.00 (0.79–1.28)	0.977	1.05 (0.68–1.62)	0.821	1.03 (0.44–2.44)	0.943
* Depression*						
Yes (ref: No depression)	1.21 (0.90–1.63)	0.212	1.71 (1.05–2.82)	0.032	0.42 (0.17–1.04)	0.060
**Increased alcohol consumption** Yes (ref: decrease/no change in alcohol consumption)	2.18 (1.54–3.07)	< 0.001				
**Increased use of psychoactive substances** Yes (ref: decrease/no change in psychoactive substance use)	3.67 (2.01–6.73)	< 0.001				

The model was adjusted for age, sex at birth, HIV status, highest level of education attained and employment status

*AOR* adjusted odds ratio

*CI* confidence interval

Table 3 shows that the odds of physical distancing was significantly lower for those who reported increased alcohol consumption (AOR = 0.60; 95%CI: 0.41, 0.89; *p* = 0.011).

**Table 3:** Multivariable logistic regression analyses for the association between COVID-19 preventive measures, increased alcohol use and increased use of other psychoactive substances during the COVID-19 pandemic by adults in Nigeria (*N* = 3160)

Variables	COVID-19 preventive measures					
	**Face masks**		**Washing of hands/sanitizing hands**		**Physical distancing**	
	**AOR** **(95% CI)**	***p* value**	**AOR** **(95% CI)**	***p* value**	**AOR** **(95% CI)**	***p* value**
**Increased alcohol consumption** Yes (ref: decrease/no change in alcohol consumption)	1.41 (0.86–2.32)	0.175	0.081 (0.50–1.31)	0.391	0.60 (0.41–0.89)	0.011
**Increased use of psychoactive substances** Yes (ref: decrease/no change in psychoactive substance use)	0.44 (0.17–1.12)	0.084	1.82 (0.71–4.65)	0.214	1.64 (0.79–3.40)	0.185

The model was adjusted for age, sex at birth, HIV status, highest level of education attained and employment status

*AOR* adjusted odds ratio

*CI* confidence interval

## Discussion

The study findings demonstrate a complex relationship between sexual health, mental health and COVID-19 behavior during the first wave of the COVID-19 pandemic. Findings indicated that increased alcohol consumption and increased use of psychoactive substances were associated with higher odds of reporting increased sexual activity. Experiencing PTSD and depression were associated with higher odds of increased alcohol consumption. Not practicing physical distancing was associated with a greater likelihood of increased alcohol consumption. The study hypotheses were therefore, partially supported.

One of the strengths of this study is the large sample size. It is also one of the few empirical studies on the association between mental health and COVID-19 in sub-Saharan Africa, and one of the few studies, if any, that show the linkages between mental health, sexual behavior, and COVID-19 preventive measures during the early phase of the pandemic in Nigeria. Identifying common factors associated with mental health, sexual health, and COVID-19-related behaviors may help with the design of interventions to reduce these risks using a common-risk factor approach.

The interpretation of the study findings is, however, limited by the cross-sectional study design which can neither determine the direction of relationships, nor prove causal relationships. The sample is also a convenient sample skewed towards participants with tertiary education. This limits the generalizability of study findings to broader populations. Also, although biological factors such as sleep, are known to be associated with sexual function [63, 64], this study did not include biological factors in the analysis but limited exploration of variables to mental health related factors. Our measure of sexual behavior described self-reported changes in sexual activities and did not measure specific behaviors or indicators of sexual dysfunction. Also, the measures for anxiety and depression were assessed through a respondent-rated, single-item question. Single item measures of depression have been found to be highly specific though and appropriate for ruling out cases of depression. The low sensitivity may however imply an underestimation of the cases of depression in this study cohort [65]. Despite these limitations, the study was able to generate hypotheses that can be further tested.

First, while sexual activity can be therapeutic [66], the observed increase associated with increased alcohol consumption and increased use of psychoactive substances during the pandemic may be troubling. Increased alcohol consumption and the use of some psychoactive substances like marijuana [67]—a common psychoactive substance used in Nigeria [68, 69]—are associated with arousal, desire, responsiveness, and loss of inhibition [70] and increase in risky sexual behaviors [71]. Risky sexual behaviors such as unprotected sex and having multiple and casual sex partners [72–75] increase the risk for sexually transmitted infections and unwanted pregnancies [76–78]. Thus, harm reduction strategies, such as promoting condom use, may be particularly relevant for those who experienced increased use of psychoactive substances, including increased alcohol consumption, during the pandemic.

Third, more worrisome is the observed association between an increase in the use of psychoactive substances and the experiences of PTSD and depression. Alcohol consumption and use of psychoactive substances can lead to high-risk outcomes. Individuals may perceive greater personal control over SARS-CoV-2 infection due to difficulties in emotion regulation and impulsivity [79, 80]. The reduced practice of physical distancing by participants who increased their consumption of alcohol in the current study may reflect poor decision-making related to the risk for contracting COVID-19. Alcohol can cause individuals to overcome caution associated with novel social spaces and promote seeking proximity to strangers with implications for spreading the virus [81–84].

Finally, from the study findings, we postulate that the anxiety, depression, and PTSD during the COVID-19 pandemic may have led to a decrease or no change in sexual activity. This decrease in sexual activity may be due to a variety of factors. Possible causes might be related to erectile dysfunction [85], increased level of stress due to increases in home chores [86, 87], anxiety-induced reduction due to concerns about contracting COVID-19 [88], or avoidance and negative alterations in cognition and mood among those who experienced PTSD [89]. The current study finding suggests that increased alcohol consumption and increased use of psychoactive substances may also have increased sexual activity.

## Conclusion

Poor mental health was associated with decreased/no change in sexual activity during the first phase of the COVID-19 pandemic in Nigeria. Depression was associated with increased consumption of alcohol and increased use of other psychoactive substance, and the use of these psychoactive substances was associated with increased high-risk sexual activity. Increased consumption of alcohol was also associated with poor practice of physical distancing during the pandemic. Future studies should explore the complex relationships between mental health, sexual activity, psychoactive substance use and the use of behavior dependent preventive measures to determine cost-effective combination approaches to reduce the adverse effects of disasters with the nature magnitude of the COVID-19 pandemic.

## Data Availability

The datasets used and/or analysed during the current study available from the corresponding author (Morenike Oluwatoyin Folayan) on reasonable request.

## Abbreviations

AOR: Adjusted Odds Ratio
CI: Confidence Interval
COVID-19: Corona Virus Infectious Disease 2019
HIV: Human Immunodeficiency Virus
PTSD: Post Traumatic Stress Disorder
SARS-CoV-2: Severe Acute Respiratory Syndrome Corona Virus Type 2
